# Fingolimod Real World Experience: Efficacy and Safety in Clinical Practice

**DOI:** 10.1155/2015/389360

**Published:** 2015-11-26

**Authors:** Joaquim Fonseca

**Affiliations:** ^1^University of Aveiro, 3810-193 Aveiro, Portugal; ^2^Novartis Farma SA, 2710-693 Sintra, Portugal

## Abstract

Fingolimod is a multiple sclerosis treatment licensed in Europe since 2011. Its efficacy has been demonstrated in three large phase III trials, used in the regulatory submissions throughout the world. As usual, in these trials the inclusion and exclusion criteria were designed to obtain a homogeneous population, with interchangeable characteristics in the different treatment arms. Although this is the best strategy to achieve a robust answer to the investigation question, it does not guaranty the treatment efficacy in the clinical practice, since in the real world there are concomitant treatments, comorbidities, adherence, and persistence challenges. But, to make informed treatment decision for a real life patient, we need to have evidence of the treatment efficacy, what has been called treatment effectiveness. This work aims to review fingolimod effectiveness, using, as source of information, abstracts, posters, and manuscripts. This unorthodox strategy was developed because more than half of the published experience with fingolimod is still on abstracts and posters. Only a small part of the studies reviewed are already published in peer reviewed journals. Fingolimod seems to be, at least, as effective and safe as it was on clinical trials, and with its long-term experience no new safety signals were observed.

## 1. Introduction

Nowadays a new multiple sclerosis (MS) drug usually has at least one phase II and two phase III clinical trials in order to get the required regulatory authorities' approval. But, as it is commonly accepted, clinical trials have a very homogeneous population without any big issues in terms of comorbidities and comedication. This is necessary in order to meet the trial's endpoints, in the shortest timeframe and with the smaller sample size possible. However, these perfect patients are rare in the hospitals and usually the patients who can be treated on-label have very different characteristics from the trials' population. There are also factors inherent to treatments, which may influence its application in clinical practice. The outcomes of using the drug in a real world setting may be very different from the ideal scenario that happens in clinical trials. Trial protocol forces uncommon clinical scenarios or the comparison group does not represent current standard of care; there may be biases in the patients who are eligible for a therapy or in patients who are ultimately treated with the therapy; the complexity of therapies makes them challenging to implement or procedural experience of providers influences outcomes; there may be limited availability of providers or resources or inadequate levels of reimbursement [1].

That is the reason why it is of utmost importance to share the experiences, present abstracts, posters, or communications in congresses, and publish clinical practice results. Furthermore, important drug outcomes, like resource utilization, treatment satisfaction, quality of life, or other Patient Reported Outcomes (PROs), are not usually included in the regulatory trials' outcomes. Safety issues are also a good reason to share experiences, because the causal association between very rare safety events and a drug can only be made if everyone makes an effort to report to the Market Authorization Holder. When these reports are not made, if physicians publish and present posters the information will be captured when the pharmacovigilance departments do the scientific literature review. Efficacy is the treatment result the drug can achieve in clinical trials and effectiveness is the result obtained in clinical practice. Efficiency is a third concept, which has gained a lot of importance especially in these last years, and it brings the economic factor to the equation, where the effectiveness is put in perspective with the economic aspects of using the drug in the real setting.

Fingolimod is approved in the United States of America since 2010 and in Europe since 2011. It has been used in more than 104,700 patients worldwide and it has an exposure of more than 195,000 patient-years [2]. I think it is important now to take a moment to reflect how the drug has been used in clinical practice and what the efficacy outcomes are.

## 2. Methods

A literature search was done to identify 2013 and 2014 congress abstracts and posters; and published manuscripts (from January 1, 2011, to September 4 15 October 2014) about fingolimod use in the clinical practice. The databases used were Medline, Embase, and Biosis previews. Case reports and studies with small samples (less than 8 patients) were excluded as well as publications with data from blinded clinical trials, because they were not considered real world data.

To avoid considering duplicate data an effort has been made to only include in the analysis the latest report of each series of patients excluding older abstracts and manuscripts with the same pool of patients and outcome measures. Whenever there was an available abstract and a published paper the information was extracted from the later. Reviews and manuscripts or abstracts reporting subgroup analysis with the same outcomes of the original study were also excluded.

For the safety data analysis, in order to avoid overestimating adverse events incidence due to small samples, a cut-off of 500 patients in the cohort was used. In order to have a broader perception of fingolimod safety, it was decided to include in the analysis the five-year interim results of the LONGTERMS extension study. The search was limited to references published after January 1, 2013.

The selection process finished with 97 unique publications, 88 of them used for the efficacy review, 3 for the safety review, and 6 for both reviews.

## 3. Results

### 3.1. Baseline Characteristics

The number of patients is the only baseline characteristic that is reported by virtually every study. Regarding multiple sclerosis outcome measures, a big proportion of studies report disability and relapses, but only 6.3% publish MRI baseline data. Almost half of the studies reported if natalizumab was the previous treatment and, within those studies, around 55% just mention the percentage of patients previously treated with natalizumab. A table with the baseline characteristics is available in Supplementary Material available online at http://dx.doi.org/10.1155/2015/389360.

Patients in the TRANSFORMS [3] study had, at baseline, mean disease duration of 7.5 years, with 1.5 relapses in previous 12 months (2.3 in previous 24 months) and EDSS of 2.24 and 67.4% of patients did not have T1Gd+ lesions on the MRI. The average patient enrolled in these studies had longer disease duration and higher EDSS score, but a lower Annualized Relapse Rate (ARR).

### 3.2. Relapse Outcomes

**Figure 1:**
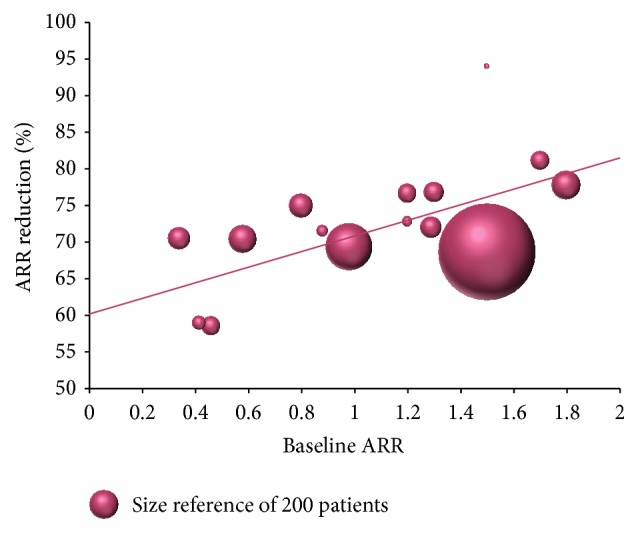
ARR reduction. The bubble size represents the patient numbers.

Relapse outcomes were reported in 48.8% of studies, which enrolled 8,483 patients. Generally, the vast majority of studies confirmed fingolimod efficacy in reducing relapse activity seen in pivotal trials. The ARR reduction weighted average was 70.1%, and the reported ARR reduction was superior to 68% in all but two studies (Figure 1).

In the studies where natalizumab was used as control, fingolimod seems to be at least as effective as natalizumab when used in second-line therapy. Fingolimod reported similar or superior results in 50% of the studies. When the control was glatiramer acetate, interferon, or dimethyl fumarate (DMF), fingolimod always showed superior relapse outcome results.

### 3.3. Disability

It is pretty difficult to summarize disability outcomes in the 29.5% of studies that reported it. The main reason is the nonstandardized approach of how this should be reported.

A total of 13 studies with 5,249 patients reported proportion of patients free of disability progression, and fingolimod achieved a weighted average of 89.9% after 13.2 months. In the 15 studies that reported EDSS difference from baseline, involving 5,212 patients, the weighted average difference in the EDSS score compared to the baseline was −0.07, and a similar result is obtained if we exclude the natalizumab switch studies.

### 3.4. MRI Outcomes

MRI outcomes were reported by 16.8% of the studies reviewed. However these studies combined represent only 4.6% of total patients. A weighted average of 91.6% of patients was free of T1Gd+ lesions and 73.6% of them were free of new T2 lesions. These numbers are consistent or slightly better than the results of the clinical trials. In TRANSFORMS [3], after one year of treatment, 90.1% patients were free of T1Gd+ lesions and 54.8% of them were free of new or enlarged T2 lesions. In FREEDOMS and its extension, after 4 years of fingolimod treatment 79.5% patients were free of T1Gd+ lesions and 80.9% of them were free of new or enlarged T2 lesions.

Worth mentioning is a 50-patient study, from Puerto Rico [4], where the number of T1 hypointense lesions and brain atrophy were evaluated. It was reported that 86% patients were free of new black holes and 90% free of brain volume loss after one year of fingolimod treatment.

Another study [5], with 30 patients, evaluated the cortical and subcortical volume changes. This study suggests that fingolimod treatment preserves the structural thickness in the majority of the brain areas over one year of treatment.

### 3.5. Composed Outcomes

In the last years MS outcome measures have evolved from evaluating only the clinical outcomes, EDSS, and relapses to focusing on the patient as whole and thus considering all 4 measures of Non-Evidence of Disease Activity-4 (NEDA-4). A patient achieves NEDA-4 when there are no relapses, no MRI activity, no disability progression, and no pathological brain volume loss. This should be the ultimate goal of each and every MS treatment.

Only 13 studies, representing 4.9% of the patients, have reported composed outcomes. Due to the low number of studies reporting most of the composed outcomes, these results must be interpreted with caution (Table 1).

Only two of the composed outcome measures reported can be compared with data from the clinical trials (Table 2). With the exception of free of EDSS progression and MRI activity, which was only reported by one study, fingolimod achieved a good proportion of patients free of activity in all the other composed outcomes. Almost half of these studies reported the proportion of patients free of clinical activity (relapse and EDSS progression).

### 3.6. Patient and Neurologist Reported Outcomes, First-Dose Observation, and Other Outcomes

#### 3.6.1. Persistence

Persistence and adherence are very important factors to the effectiveness of any treatment, because even with a very efficacious treatment it is of no use if the patients discontinue treatment or do not adhere to the recommended posology.

Persistence/adherence was reported by 46.3% of the studies, which observed a total of 18,471 patients. To calculate the weighted average, 8 studies were excluded either because the persistence was not reported in a way compatible with the calculation or because the study did not report the follow-up period. The weighted average, considering both patient numbers and follow-up duration, was 82.8%, after 11.6 months of follow-up.

#### 3.6.2. Patient and Neurologist Reported Outcomes (PRO/NRO)

The impact of a chronic, debilitating disease as MS goes beyond its impact on patient's physiology and is of utmost importance to give the deserved relevance to other outcomes that are also important, like quality of life, treatment satisfaction, work productivity, cognition, and impact on family and care givers. Unfortunately most pivotal trials do not assess this outcome and that is where real world evidence can give an important contribution to the body of knowledge.

Montalban et al. [6] published one study about the effect of treatment in quality of life and depression in the phase II fingolimod trial. This study suggests that with fingolimod a smaller proportion of patients present signs of clinical depression than with placebo (*p* = 0.0018), even though at baseline there were more fingolimod patients with signs of clinical depression. The 24-month extension of this study suggests that this effect can be observed in the first 6 months of treatment, and after that the depression symptoms are stabilized until month 24.

Generally speaking fingolimod has a positive impact on patients' lives, in the domains reported by these studies: quality of life, cognition, tolerability, depression, treatment satisfaction, and fatigue (Table 3). In these 19 studies, which reported PRO/NRO, a total of 6,905 patients were followed up, but each study did not report all the PRO/NRO dimensions. For instance, QoL was reported in 7 studies, which followed up 6,136 patients. On the other hand, only 1 study following up 790 patients reported fatigue outcomes. Given the great impact these domains have in patients' lives it is desirable that in the future even more studies look into this.

#### 3.6.3. First-Dose Observation

The transient cardiovascular effects of the first fingolimod dose were reported by 25.3% of the studies, which enrolled 16,683 patients. The first dose of fingolimod was uneventful in a big proportion of patients, usually >90%, and most of the patients (≈95%) were discharged after the 6-hour observation period.

FIRST [7] was a 2,417 patient open-label multicentre study, sponsored by Novartis, to assess the overall short-term safety and tolerability of fingolimod 0.5 mg. This study enrolled 12.2% of patients with cardiac risk and 5% treated with calcium channel blockers and/or beta blockers. A Mobitz type I second-degree atrioventricular block was found in 4.1% patients before the first-dose administration. In the group without cardiac risk there was an incidence of new events after dose of 1.1% and this incidence was 4.0% in the group with cardiac risk.

An Italian study [8] with 112 patients found that 20.1% patients showed abnormalities in the baseline ECG, including a first-degree atrioventricular block in 0.9% of patients. There was 1.8% of* de novo* first-degree AV block and 0.9% of* de novo* second-degree AV block.

#### 3.6.4. Other Outcomes

A 12-month follow-up study of 55 Italian patients reported that active patients had more relapses before fingolimod (*p* = 0.043), a lower mean white blood cell count (*p* = 0.027), and a lower mean lymphocyte count (*p* = 0.05) compared to disease-free patients. The authors' hypothesis is that “protective” cell subsets may be also affected by the excessive lymphopenia [9].

One Italian study, with 35 patients, focused on fingolimod effect on headache and reported 2.9% of* de novo* headache. Headache was reported in 23–25% of patients in the phase III clinical trials, but there is no reference to the baseline incidence [10].

Retinal and macular volume effects of fingolimod were the focus of two American studies, with 232 and 126 patients [11, 12]. In Cleveland clinical study it was not possible to detect a change in macular volume, but a small increase in central subfoveal thickness and outer retina thickness was detected. The study from Seattle reported a decrease in macular volume (*p* = 0.003). Both studies reported a trend of ganglion cell/internal plexiform layer and retinal nerve fibre layer thinning. It can be speculated that these changes may be disease and/or treatment related; thus further studies with a control arm would be helpful to clarify how much these effects can be attributable to fingolimod.

#### 3.6.5. Natalizumab Switch

Given that natalizumab patients have a risk of developing Progressive Multifocal Leukoencephalopathy (PML), especially after 24 months of treatment [13], it is common to switch these patients to fingolimod, as a therapy of similar efficacy. Natalizumab patients are usually in a more advanced state of disease than patients previously treated with first-line DMTs. So it is expectable to get results that are not as good as when fingolimod is used in a DMT switch population.

Around 20% of studies focused on the natalizumab switch strategy. Many strategies have been employed and the review of these studies suggests that the switch to fingolimod is the most successful strategy, even though it is not in this population that we can observe fingolimod's best results.

EDSS values greater than 3, washout period length, and ARR (before treatment and during natalizumab treatment) have been proposed as predictors for relapses after natalizumab, but probably due to the small samples, it has been impossible to achieve significant results across several studies. The washout period length seems to be the most consensual predictor.

The studies reviewed suggest that in a significant number of patients after natalizumab interruption disease activity goes to prenatalizumab levels in the first 6 months. The majority of studies recommend a maximum washout period of 6 to 12 weeks. In most studies, a shorter washout was associated with a lower risk of disease reactivation. Fingolimod in these studies has been shown to be able to control disease reactivation in most patients. It can be speculated that patients previously treated with natalizumab are, for obvious reasons, in a more advanced disease level, when the diffuse damage starts to be relatively more important than the focal damage, even though the two types of damage coexist. This hypothesis could explain both the inefficacy of first-line DMTs, which only act on the inflammation (focal damage), and the efficacy of fingolimod, which has a double mechanism of action, acting upon the immune system, reducing the focal damage, and directly in the central nervous system reducing the diffuse damage.

In this population fingolimod seems to avoid a reactivation of disease in most patients.

#### 3.6.6. Safety Outcomes

Analysing real world evidence for safety outcomes is difficult, because most of the studies have less than 500 patients, and thus incidence rate of rare events can be unrealistically overestimated. A cut-off of 500 patients in each study was used to avoid this artefact. This resulted in a substantial drop of the number of studies available to review. On the other hand, a big proportion of real world studies suffer an underreporting bias because physicians tend not to report AEs if they are not serious and are described in the Summary of Product Characteristics. The dimension of this problem seems to be inversely proportional to the sample size of the study. It is usual that studies with small samples do not report any AE. If all the studies were included in this analysis it would result in an artificially low incidence of most AEs.

Safety issues, even more than efficacy outcomes, suffer from a great variability in the way they are reported. In the 7 cohorts analysed, 145 different adverse events (AEs) were identified, but some appear twice in the list if in one study they were classified as AE and in another study they were classified as Serious Adverse Events (SAEs). In 77.9% of the AEs/SAEs, the incidence reported in the clinical trials was superior or similar to the reported incidence in the studies reviewed. Generally, the real world practice confirms the safety profile already established in the clinical trials.

In the first dose of fingolimod a short-term and asymptomatic heart rate decrease can be observed in most cases. The studies reviewed confirm the findings in the clinical trials for the cardiovascular AEs [14, 15], with only a small number of events, reported in more than one of these studies, being unheard before.

Asymptomatic ALT and bilirubin increases were reported mostly in the first months of fingolimod treatment in clinical trials. After treatment interruption, the enzymes returned to normal levels within 6 months [14, 16]. Once more, the different ways of reporting these AEs make it difficult to make a comparison between studies.

Macular oedema was reported in 0.6% patients in clinical trials [14], mostly in the first 6 months of treatment, and in most cases with recovery after treatment interruption without the need of macular oedema treatment. The macular oedema incidence reported in all studies was 0.32%, and it was calculated by dividing the number of reports in the study by the total population enrolled in the reviewed studies for safety outcomes.

Fingolimod overall incidence of infections and serious infections was similar to placebo in clinical trials, with lower respiratory tract infections being the only type of infections with slightly higher incidence than placebo [14]. The reviewed studies confirmed this infection profile. Worth mentioning are the incidences in all studies (calculated by dividing the number of reports in the study, by the total population enrolled in the reviewed studies for safety outcomes) of reactivation of chronic viral infections (0.79%) and herpes zoster infections (0.25% versus 3.1% reported in clinical trials [14]).

Fingolimod mechanism of action involves the selective and reversible retention of lymphocytes in secondary lymphoid organs, thus having an impact in the serum quantification of these cells. Because the different studies may have used different thresholds and ways of reporting, it is useful to present these differences in Table 4. In clinical trials 73% average decrease in lymphocyte count compared to baseline was reported and lymphopenia was reported as an AE in 12% of patients [14]. Leukopenia was reported in 2.82% of patients in FREEDOMS [17] and 1.6% in TRANSFORMS [3].

Incidence of malignancies in clinical trials was similar in the fingolimod 0.5 mg and in the placebo arms. In clinical trials, the Basal Cell Carcinoma (BCC) showed a trend towards a bigger incidence with fingolimod 0.5 mg than with placebo [14]. Two studies reported only skin cancer, whereas in clinical trials skin cancers were classified as BCC or Squamous Cell Carcinoma (SCC). The ratio BCC/SCC is similar to that observed in general population [18] and different from that observed in immunosuppressed populations [19].

## 4. Discussion

This review tried to gather as much real world data as possible and thus very inclusive criteria were used to select the studies to be reviewed. Clinical trials have a very homogeneous population, which guarantees high precision of the results and high internal validity. This strategy results, most of the time, in studies with a less than perfect external validity. On the other hand, studies in clinical practice setting usually have a more heterogeneous population, resulting in a lower internal validity, but with a higher external validity. My belief is that when considered individually these studies cannot be used to reach any conclusion about the treatment effectiveness due mostly to small samples and when considered as a whole they become more robust and credible to be used to reach drug effectiveness deductions.

In this work, references in languages other than Portuguese, English, Spanish, and French were not reviewed. Posters and abstracts that were not available online or that were not seen in the respective congress were naturally excluded from this review. Regarding oral presentation only its published online abstracts have been considered. Even with all these exclusions, a big proportion of RWE data shared by clinicians in congresses and peer reviewed journals was captured.

Fingolimod has proven in an extensive clinical development program its effectiveness in reducing relapse rate, disability progression, MRI activity, and brain volume loss. In FREEDOMS, after 2 years, the likelihood of achieving Non-Evidence of Disease Activity in its 4 parameters with fingolimod was more than 4-fold higher versus placebo.

In clinical practice, patients are not as perfect and homogeneous as in clinical trials; they have comorbidities, concomitant medications, and other idiosyncrasies. Real world evidence is very important to answer questions for which clinical trials have not looked for the answers and to assess treatments' effectiveness in clinical practice.

The ultimate goal of this review, to have an overview of fingolimod effectiveness in clinical practice, was achieved. The results suggest that fingolimod treatment is initiated in a later phase than in clinical trials, in patients with more disease duration and higher EDSS score, even though with a lower ARR. The relapse outcomes achieved with fingolimod are pretty much in line of those obtained in clinical trials, with an ARR reduction usually above 70%. Fingolimod achieved 90% of patients without disability progression after 13.2 months of follow-up. In the MRI parameters, 91.6% patients were free of T1Gd+ lesions and 73.6% of them were free of new T2 lesions, which is a result slightly better than what was seen in clinical trials. Unfortunately a very low number of studies reported composed outcomes, but what was reported suggests that fingolimod has better results than in clinical trials, having achieved 62.5% patients free of relapse, EDSS progression, and MRI activity in three studies and 66.6% patients free of relapse and EDDS progression in 7 studies.

In these studies, after 11.6 months 82.6% of patients continue on treatment and fingolimod showed a positive impact in patients' quality of life, cognition, tolerability, depression, treatment satisfaction, and fatigue. The first-dose observation results presented no surprises, just confirming what was seen in clinical trials. It was uneventful in a big proportion of patients, usually >90%, and most of the patients (≈95%) were discharged after the 6-hour observation period.

Fingolimod has demonstrated superiority in all studies where interferon and/or glatiramer acetate was the control, and with DMF, even though, in the last case, the study is too short to reach any reliable conclusion, fingolimod was more efficacious. In the studies where natalizumab was used as control, fingolimod seems to be at least as effective as natalizumab when used in second-line therapy. Fingolimod reported similar or superior results in 50% of the studies. It is hard to reach any firm conclusion because only 25% of these studies reported the proportion of patients who had natalizumab as previous treatment, which in two of these studies was as high as 50%.

In the natalizumab switch studies fingolimod seems to avoid a reactivation of disease in most patients. And it seems clear that a short natalizumab washout is associated with better outcomes. The majority of studies recommend a maximum washout period of 6 to 12 weeks, and I have attended oral communications where 0- to 4-week washout was recommended.

## 5. Conclusion

These studies suggest that fingolimod is effective and well tolerated and has a safety profile that is very similar to what was observed on clinical trials. No new safety signals have been identified.

## Highlights

- 97 unique publications were selected, 88 of them used for the efficacy review, 3 for the safety review and 6 for both reviews.
- Fingolimod's real world experience seems to confirm that fingolimod is effective and well tolerated and with a safety profile that is very similar to what was observed on clinical trials.

## Supplementary Material

This table summarizes the baseline characteristics of the studied population and identifies each study by country, sample size and cohort name.

## Figures and Tables

**Table 1:** Weighted average of composed outcomes reported.

	WA	Max	Min	Nr	Pts
Patients	117	306	21	13	1515
Patients with NTZ as previous treatment	28,2%	100,0%	15,3%	7	741
Baseline ARR (previous 12 months)	0,64	1,20	0,34	6	606
Baseline MRI activity	78%	78%		1	100
Baseline EDSS	2,4	3,9	2,3	6	572
FU (months)	11,96	16,30	6,00	12	1494
Free of relapse and T1Gd+	**93,5%**	97,3%	89,1%	2	141
Free of EDDS progression and MRI activity	**42%**	42%	42%	1	37
Free of relapse and MRI activity	**68,8%**	87,6%	47,6%	3	504
Free of relapse and EDSS progression	**66,6%**	79,7%	56,3%	7	770
Free of relapse, EDDS progression, and MRI activity	**62,5%**	72,5%	50,3%	3	419

WA: weighted average; Max: maximum; Min: minimum; Nr: number of studies reported; Pts: number of patients.

**Table 2:** Composed outcomes of pivotal trials.

	FREEDOMS 2 years	TRANSFORMS 1 year
Patients	425	429
Mean follow-up (months)	24	12
Baseline ARR (previous 12 months)	1.5	1.5
Baseline EDSS	2.24	2.2
Free of relapse and EDSS progression	**62%**	**79%**
Free of relapse, EDDS progression, and MRI activity	**33%**	**46%**

**Table 3:** Patient and neurologist reported outcomes summary.

Dimension	Patients	Studies	Results		
			Positive	Neutral	Negative
Cognition	711	2	2	0	0
Depression	1,609	7	5	2	0
Fatigue	790	1	1	0	0
QoL	6,136	7	5	2	0
Tolerability	4,021	2	2	0	0
Treatment satisfaction	1,382	4	4	0	0

QoL: quality of life.

**Table 4:** Leukocyte and lymphocytes count changes.

AE	Incidence in individual studies	Incidence in all studies
Leukopenia and lymphopenia	6.9%	1.03%
Lymphocyte count decreased	0.3%; 1.24%; 3.8%; 2.1%; 2.3%	1.21%
Lymphopenia	6.5%	1.23%
White blood cell count decreased	8.3%	0.3%

Incidence in all studies was calculated by dividing the number of reports by the total population enrolled in the reviewed studies for safety outcomes.

## Abbreviations

MS: Multiple sclerosis
CNS: Central nervous system
EDSS: Expanded Disability Status Scale
RRMS: Relapsing-Remitting Multiple Sclerosis
PROs: Patient Reported Outcomes
MRI: Magnetic resonance imaging
QoL: Quality of life
DMT: Disease-modifying therapy
ARR: Annualized Relapse Rate
DMF: Dimethyl fumarate
NEDA-4: Four parameters of Non-Evidence of Disease Activity
FDO: First-dose observation
PML: Progressive Multifocal Leukoencephalopathy
AEs: Adverse events
SAEs: Serious Adverse Events
BCC: Basal Cell Carcinoma
SCC: Squamous Cell Carcinoma.
