# Supplementary material for: Fingolimod Real World Experience: Efficacy and Safety in Clinical Practice
Source: Neurosci J. 2015 Nov 26;2015:389360. doi: 10.1155/2015/389360 (PMC4674597; doi:10.1155/2015/389360)
Supplement: Supplementary file 1 — This table summarizes the baseline characteristics of the studied population and identifies each study by country, sample size and cohort name. [file 389360.f1.pdf]

Table 1. Baseline characteristics

Cohort	Country	Focus	Patients	Time since diagnosis of MS (years)	Patients with NTZ as previous treatment	Washout period from NTZ (weeks)	NTZ infusions	Baseline ARR (previous 12 months)	Baseline MRI activity	Baseline EDSS	Source of data	FU (months)
REAL[1]	Argentina	Registry	48	9.1						2.7	Reg	8.5
Multiple Sclerosis Treatment Registry[2]	Austria	FTY vs NTZ	312 (NTZ 1208)	8.9 (NTZ 7.8)	54.2%			1.8 (NTZ 2.3)		2.6 (NTZ 3.2)	Reg	21.5 (NTZ 35.7)
São Paulo[3]	Brazil	FDO	180								R	
Gilenya* Go Program(TM)[4]	Canada	PRO	1700								P	
Multiple Sclerosis Registry Denmark[5]	Denmark	FDO	496	10.6	32.3%			0.37 (PNTZ patients 0.46)			R	> 3
Alsace[6]	France	NTZ switch	59		100%	6.8					R	7
Alsace[7]	France	Efficacy	290							3.6	R	12
Bordeaux[8]	France	FDO	24								P	3
ENIGM[9]	France	NTZ switch	333		100%	17 (55% patients without MP or other DMTs)	31			3.7 (pre-NTZ 3.6; post-NTZ 3.6)	P	6
GRACE[10]	France	PRO	198	8.1				1.6		2.6	P	
Reims[11]	France	Efficacy	66								P	11 (51.5% >12 months)
Dresden[12]	Germany	FDO	36	4							P*	
Munich, Bochum, Marburg[13]	Germany	NTZ switch	33		100%		31.8	0.2 (pre-NTZ 2.5)		3.2 (pre-NTZ 3.1)	R	20.3
NeuroTransConcept[14]	Germany	FTY vs NTZ	190 (NTZ 239)	9.89 (NTZ 9.15)				0.34 (NTZ 0.42)		2.3 (NTZ 3.3)	R	12
PANGAEA[15,16,17]	Germany	Large open trials	3641 (PEARL active 457; PEARL inactive 1248)	8.2 (PEARL active 6.7; PEARL inactive 7.3)	18.4%	31.9% Patients with WO <12		1.5 (PNTZ 0.9; PEARL active 1.23; PEARL inactive 0.37)		3 (PEARL active 2.60; PEARL inactive 2.20)	P	<24
START[18]	Germany	FDO	1640							2.8	P	
Athens[19]	Greece	NTZ switch	60		33.3%						NA	>12 (MP 8-12)
Ramat-Gann[20]	Israel	Efficacy	110	14.6						3.8	R	20
Tel-Aviv[21,22]	Israel	Other	30					1.2		0.4 increase in previous 12 months	P	12

Cohort	Country	Focus	Patients	Time since diagnosis of MS (years)	Patients with NTZ as previous treatment	Washout period from NTZ (weeks)	NTZ infusions	Baseline ARR (previous 12 months)	Baseline MRI activity	Baseline EDSS	Source of data	FU (months)
Ferrara, Parma Modena, Reggio-Emilia, Fidenza and Piacenza[23]	Italy	Efficacy	127	10.7	30.7%	18		1.2	65,4% (31.5% T1Gd+)	3.1 (PNTZ patients 3.6)	P*	10
Gallarate[24]	Italy	Other	55	12.5	21.8%			1.7 in previous 24 months			NA	12
Gallarate[25]	Italy	NTZ switch	32		100%	15.6	21				NA	73.3% patients with >6 months
iMedWeb registry[26]	Italy	NTZ switch	106 (DMTs 106)	13.15 (DMTs 12.83)	100%		26.41 (DMTs 19.42)			3.79 (DMT 3.95)	R	
L' Aquila, Pozzilli, Foggia[27]	Italy	Efficacy	142		19.7%						NA	15
L' Aquila, Pozzilli, Foggia[28]	Italy	FTY vs NTZ	100 (NTZ 100)					1.2 (NTZ 1.9)	78% of patients (NTZ 90%)	2.6 (NTZ 2.9)	P	12
Milan[29]	Italy	NTZ switch	61		100%	<52		1.2 (0.15 on NTZ; 1.2 WO period)		EDSS increase 0.43 (0.15 on NTZ; 0.43 WO period)	R*	>6
Milan[30]	Italy	FTY vs NTZ	37 (NTZ 160)	11.5 (NTZ 10.1)				1.2 (NTZ 1.3)		2.1 (NTZ 2.5)	P	>12
Multicenter Italy[31]	Italy	FDO	906	8.56	24.3%			1.36		3.06	P	3.14
Naples[32]	Italy	FDO	112								P*	
Orbassano[33]	Italy	NTZ switch	35 (T 19; NT 24)			>12					R	
Padua[34]	Italy	NTZ switch	21	10.7	100%	12				2.9	P*	
Rome[35]	Italy	FDO	55							1.7	P	
Turin[36]	Italy	Other	35	11.3							P	6
Multicenter Japan[37]	Japan	Registry	837	9.3				0.98			Reg	
Tokyo[38]	Japan	Efficacy	44	13.1	0%						R	
Kuwait city[39]	Kuwait	Efficacy	76	7.82	2.6%			13.2% relapse free	22.4% MRI activity free	2.93	R	18.5
MS registry Kuwait[40]	Kuwait	Efficacy	175	7.2	5.7%			32.6% relapse free	23.3% MRI activity free	2.6	Reg	21.7
FIRST[41]	Multinational	FDO	2417		10.5%			1.1		2.4	P	4
LONGTERMS[42,43]	Multinational	Safety									P	

Cohort	Country	Focus	Patients	Time since diagnosis of MS (years)	Patients with NTZ as previous treatment	Washout period from NTZ (weeks)	NTZ infusions	Baseline ARR (previous 12 months)	Baseline MRI activity	Baseline EDSS	Source of data	FU (months)
MSBase Registry[44]	Multinational	NTZ switch	89	12.8	100%	95% with WO < 4 months; 2.6 months		0.26		4	R	7.6
MSBase Registry[45]	Multinational	FTY vs NTZ	171 (NTZ 407)	9.5 (NTZ 9.4)				1.29 (NTZ 1.53)		3 (NTZ 3.5)	P	12
MSBase Registry[46]	Multinational	FTY vs DMTs	148 (DMTs 379)	7.2 (DMTs 7.3)				1.3 (DMT 1.2)		2.5 (DMT 2.5)	P	11
PASSAGE[47]	Multinational	Large open trials	1596	8.56	19.6%			1.0		2.72	P	9
Warsaw[48]	Poland	Efficacy	11	>3							NA	12
Coimbra[49]	Portugal	NTZ switch	29	10.4	100%	9.2	28.7	0.5		2.7	R	17.4
Guaynabo[50]	Puerto Rico	Efficacy	50								R	12
Guaynabo[51]	Puerto Rico	FDO	25								R	6
Moscow[52]	Russia	Efficacy	11								NA	
Prešov[53]	Slovak Republic	NTZ switch	9		100%	3.3	24.1	0 (pre-NTZ >2)			NA	
Alicante[54]	Spain	NTZ switch	8	11.1	100%	12	29.4	pre-NTZ 1.6; pre-FTY 0.13;		2.4	P*	9.35
Granada[55]	Spain	FDO	42	9							R	
Hospital Ntra Sra Valme, Seville[56]	Spain	NTZ switch	49		100%		>12	59.2% free of relapses			NA	
Hospital Universitario Virgen Macarena, Seville[57]	Spain	Efficacy	47		36.5%			0.88 previous 24 months		3.89	R	16.3
Multicenter1 Spain[58]	Spain	FDO	148	9.1							Reg	6.1
Multicenter2 Spain[59]	Spain	Registry	267	9.3	27%						Reg	13.1
Murcia[60]	Spain	NTZ switch	12	9.1	100%	12 (with MP)	31	0.3		3.2	NA	15.4
Murcia[61]	Spain	FDO	18	7.5							P	
Santiago de Compostela[62]	Spain	Efficacy	101	9.54	30%			2.04			R	
Valencia[63]	Spain	NTZ switch	11	6.8	100%	8	37.9	pre-NTZ 1.5		3.5	P	11.7
MS registry Sweden[64]	Sweden	FTY vs NTZ	628 (NTZ 447; NTZ-naïve 306)	7.3 (NTZ 4.5; NTZ-naïve 6.4)	48.1%					2.63 (NTZ 2.35; NTZ-naïve 2.48)	Reg	>12
Multicenter Sweden[65]	Sweden	Registry	674 (186 with 12month FU)		33%						Reg	12

Cohort	Country	Focus	Patients	Time since diagnosis of MS (years)	Patients with NTZ as previous treatment	Washout period from NTZ (weeks)	NTZ infusions	Baseline ARR (previous 12 months)	Baseline MRI activity	Baseline EDSS	Source of data	FU (months)
Basel[66]	Switzerland	NTZ switch	15	11.8	100%	8	41.4	0.4	100% free of T1Gd+	3.5	P*	8
SWISSASCENT[67]	Switzerland	PRO	212	6.6	28%			0.8		2.1	P	8.3
Istanbul[68]	Turkey	Efficacy	109	9.0				1.9		3.5	P	5.5
Wimbledon[69]	UK	FDO	143								P*	
Boston[70,71]	USA	Efficacy	177	11	15.3%			0.5		1.9	R	>12
Boston[72]	USA	FDO	305								R	
Cleveland Clinic[73]	USA	FDO	317	12.1	11.6%						R	3.3
Cleveland Clinic[74]	USA	Efficacy	306								R*	11.1
Cleveland Clinic[75]	USA	Efficacy	196	11.5							R	8.6
Cleveland Clinic[76]	USA	Other	232	10.5							P	6.4
Cleveland Clinic[77]	USA	FTY vs DMF	317 (DMF 426)	14.1 (DMF 12.3)	12.4% (DMF 11.7%)						R	3
Commercial health plan or Part D program[78]	USA	PRO									R	
Cullman[79]	USA	NTZ switch	75		100%						NA	>6
Houston[80]	USA	FDO	59								R	
Medco Health Solutions[81]	USA	PRO	248								R	12
Multicenter1 USA[82]	USA	PRO	205								P	6
Multicenter2 USA[83]	USA	PRO	380	9.8	8.4%						CS	
Multicenter3 USA[84]	USA	NRO	102 Neurologists								CS	
NARCOMS[85]	USA	PRO	50 (NTZ 50)					84% relapse free in previous 6 month (FTY and NTZ)			Reg	54 (NTZ 48)
Partners MS Center[86]	USA	FTY vs NTZ	36 (NTZ 69)	7.9 (8.4; p= 0.71)				0.92(NTZ 0.8; p= 0.38)	1.56 T1Gd+ in previous 12 months (1.39; p= 0.78)	1.75 (NTZ 1.5; p= 0.42)	R	18

Cohort	Country	Focus	Patients	Time since diagnosis of MS (years)	Patients with NTZ as previous treatment	Washout period from NTZ (weeks)	NTZ infusions	Baseline ARR (previous 12 months)	Baseline MRI activity	Baseline EDSS	Source of data	FU (months)
PharMetrics Plus™[87]	USA	PRO	889 (GA 1233; IFN 1341; NTZ 287)					58.6% relapse free (GA 68.0%; IFN 68.2%; NTZ 54.7%); ARR 0.67 (GA 0.39; IFN 0.39; NTZ 0.74)			Claims	12
PharMetrics Plus™[88]	USA	FTY vs NTZ	185 (NTZ 185)					1.56 (NTZ 1.52)			Claims	12
PharMetrics Plus™[89]	USA	FTY vs DMTs	132 (GA 132)					0.46; 66.7% relapse-free (GA 0.49; 66.7%)			Claims	12
PharMetrics Plus™[90]	USA	FTY vs DMTs	128 (DMT 397)					41.4% with >1 relapse (DMTs 15.1%; p< 0.0001); ARR 1.7 (DMTs 1.26)			Claims	18
Seattle[91]	USA	Other	126								R	7
US LRx™ and PharMetrics Plus™[92]	USA	PRO	1553 (DMF 9363)								Claims	>6
EPOC[93]	USA, Canada	FDO	976	12.1				0.8		2.4	P	6
EPOC[94]	USA, Canada	PRO	847								P	6
EPOC[95]	USA, Canada	PRO	790 (DMTs 263)	12.1 (DMT 11.7)	0.1% (DMTs 0%)			0.8 (DMT 0.8)		2.4 (DMT 2.4)	P	6
EPOC[96]	USA, Canada	PRO	790 (DMTs 263)	12.1 (DMTs 11.7)				0.8 (DMT 8.8)		2.4 (DMTs 2.4)	P	6
EPOC[97]	USA, Canada	Safety	783 (DMTs 245)								P	6

CS - Cross-Sectional; DMTs - Disease Modifying Treatments; EDSS - Expanded Disability Status Scale; FDO - First Dose Observation; FTY – Fingolimod; FU – Follow-up; MP – Methylprednisolone; MRI - Magnetic Resonance Imaging; MS – Multiple Sclerosis; NA - Non-available/unidentifiable; NT – Non treated; NTZ – Natalizumab; P – Prospective; P* - Presumably Prospective; PRO - Patient Reported Outcomes; R – Retrospective; R* - Presumably Retrospective; Reg – Registry; T – Treated; T1Gd+ - T1 Gadolinium-Enhancing Lesions; WO - Washout

References

[1] Seifer G, Kuperman G, Villa A, et al. Argentinean registry of patients with relapsing remitting multiple sclerosis treated with fingolimod (REAL): design and results of interim analysis. Mult. Scler. 2014;20(S1):165-166.

[2] Enzinger C, Leutmezer F, Kraus J, et al. Treatment MS. Real Life Use of Natalizumab and Fingolimod in Austria : Benefit-Risk Data from the Austrian Multiple Sclerosis Treatment Registry. Poster presented at the Joint ACTIRIMS-ECTRIMS Meeting, Sept 10–13, 2014, Boston, USA

[3] Fragoso YD, Arruda CC, Arruda WO, et al. The real-life experience with cardiovascular complications in the first dose of fingolimod for multiple sclerosis. Arq. Neuropsiquiatr. 2014;72(9):712-714.

[4] Freedman MS, Burton J, Schecter R. Real-life patient adherence and follow-up monitoring with fingolimod. Can. J. Neurol. Sci. 2014;41(S1):S28.

[5] Voldsgaard A, Koch-Henriksen N, Magyari M, et al. Cardiac and pulmonary effects related to initiation of fingolimod treatment. Poster presented at the ECTRIMS congress, Octob 2–5, 2013, Copenhagen, Denmark

[6] Berthe C, Ongagna J-C, Collongues N, et al. Expérience alsacienne sur l'instauration du Fingolimod après l'arrêt du Natalizumab. Rev. Neurol. (Paris). 2014;170(S):A101.

[7] Berthe C, Ongagna J, Collongues N, et al. Tolerability, safety and efficacy of fingolimod in clinical practice, experience of multiple sclerosis health network in Alsace region. Poster presented at the Joint ACTIRIMS-ECTRIMS Meeting, Sept 10–13, 2014, Boston, USA.

[8] Gary, C, Gerbouin, O, Venet, A, et al. Étude observationnelle prospective sur six mois de la prise en charge des patients sous fingolimod au CHU de Bordeaux Éducation thérapeutique des patients atteints de sclérose en plaques en réseau neuro SEP. Rev. Neurol. (Paris). 2013;169S:A105.

[9] Cohen M, Maillart E, Tourbah A, et al. Switching From Natalizumab to Fingolimod in Multiple Sclerosis A French Prospective Study. JAMANeurology 2014;71(4):1-6.

[10] Moreau T, Heinzlef O, Suchet L, et al. Amélioration de L'état D'Anxiété et Stratégies de Coping Pendant les Premiers Mois D'Initiation du Traitement par FTY Chez des Patients Présentant une Sclérose en Plaques Rémittente-Récurrente Très Active en France. Abstract presented at the Journés de Neurologie de Langue Française, April 1–4, 2014, Strasbourg, France

[11] Maitrot A. Thesis abstract: Données d'efficacité et de tolérance du traitement par fingolimod dans la sclérose en plaques rémittente récurrente. Univ. Reims Champagne-Ardenne 2013.

[12] Eisele JC, Zwierzanska J, Reimann M, *et al*. Short-term and Long-term Effects of Fingolimod on the Cardiovascular Autonomic Nervous System. *Neurology* 2014;82(10 Suppl. 1):P2.196.

[13] Hoepner R, Havla J, Eienbröker C, *et al*. Predictors for multiple sclerosis relapses after switching from natalizumab to fingolimod. *Mult. Scler.* 2014;20(13):1714-20.

[14] Braune S, Lang M, Bergmann A. Second line use of Fingolimod is as effective as Natalizumab in a German out-patient RRMS-cohort. *J. Neurol.* 2013;260(12):2981-5.

[15] Ziemssen T, Fuchs A, Schwarz HJ, *et al*. 24-month interim results of PANGAEA: A 5 year registry study evaluating long-term safety , efficacy and pharmaco-economic data of German multiple sclerosis patients on fingolimod therapy. *Poster presented at the 66th American Academy of Neurology Annual Meeting, April 26–May 3, 2014, Philadelphia, PA*

[16] Ziemssen T, Moser S, Schwarz HJ, *et al*. Comparison of efficacy and treatment satisfaction of patients on baseline therapy and fingolimod : results of the interim analysis of two German registry studies PANGAEA and PEARL. *Poster presented at the 66th American Academy of Neurology Annual Meeting, April 26–May 3, 2014, Philadelphia, PA*

[17] Ziemssen T, Fuchs A, Schwarz H, et al. PANGAEA : A 5-year non-interventional study of safety , effectiveness and pharmacoeconomic data for fingolimod patients in daily clinical practice – 24 months interim results. Poster presented at the Joint ACTIRIMS-ECTRIMS Meeting, Sept 10–13, 2014, Boston, USA

[18] Limmroth V, Hoyer S, Schmidt S, *et al*. Good cardiac safety in patients with relapsing remitting multiple sclerosis upon first fingolimod dose. *Poster Present. Jt. Poster presented at the Joint ACTIRIMS-ECTRIMS Meeting, Sept 10–13, 2014, Boston, USA*

[19] Evangelopoulos M, Andreadou E, Panagiotou M, *et al*. Follow up of multiple sclerosis patients receiving fingolimod treatment: Switching of natalizumab to treatment with fingolimod, clinical and MRI findings. *Mult. Scler.* 2013;19(11 Suppl. 1):243-244.

[20] Harai G, Dolev M, Magalashvili D, *et al*. Fingolimod treatment in clinical practice. *Mult. Scler.* 2013;19(11 Suppl. 1):270-271.

[21] Barak Y, Magalashvili D, Paz T, *et al*. Fingolimod (Gilenya) confers cognitive stability in active relapsing-remitting multiple sclerosis patients. *Poster presented at the Joint ACTIRIMS-ECTRIMS Meeting, Sept 10–13, 2014, Boston, USA*

[22] Achiron A, Hoffmann C, Nissan Y, *et al*. Cortical and subcortical volume dynamics in active relapsing-remitting multiple sclerosis patients treated with in Fingolimod (Gilenya). *Poster presented at the Joint ACTIRIMS-ECTRIMS Meeting, Sept 10–13, 2014, Boston, USA*

[23] Baldi E, Guareschi A, Vitetta F, *et al*. Previous treatment influences fingolimod efficacy in relapsing-remitting multiple sclerosis: results from an observational study. *Curr. Med. Res. Opin.* 2014;30(9):1849-55.

[24] Zaffaroni M, Baroncini D, Annovazzi P, *et al*. Blood lymphocyte count: A biomarker of fingolimod efficacy? *Mult. Scler.* 2013;19(11 Suppl. 1):499-500.

[25] Zaffaroni M, Baroncini D, Annovazzi P, *et al*. From natalizumab to fingolimod: An observational study of 32 patients. *Mult. Scler.* 2012;18(4 Suppl. 1):429-430.

[26] Iaffaldano P, Lepore V, Lucisano G, *et al*. Risk of relapse after natalizumab discontinuation: which is the best treatment option? *Poster presented at the Joint ACTIRIMS-ECTRIMS Meeting, Sept 10–13, 2014, Boston, USA*

[27] Totaro R, Costantino G, Fantozzi R, *et al*. Fingolimod Treatment In Relapsing-Remitting Multiple Sclerosis Patients: A Clinical Practice Experience. *Poster presented at the 66th American Academy of Neurology Annual Meeting, April 26–May 3, 2014, Philadelphia, PA*

[28] Totaro R, Costantino G, Fantozzi R, *et al*. Efficacy of second-line treatments in multiple sclerosis patients: a multicenter experience in clinical practice in Italy. *Poster presented at the Joint ACTIRIMS-ECTRIMS Meeting, Sept 10–13, 2014, Boston, USA*

[29] Messina M, Rodegher M, Baroncini D, *et al*. Can fingolimod treatment influence disease progression after natalizumab discontinuation? *Mult. Scler.* 2013;19(11 Suppl. 1):268-269.

[30] Baroncini D, Ghezzi A, Stefanin C, *et al*. Natalizumab versus Fingolimod in active relapsing-remitting multiple sclerosis: a prospective observational study of 197 patients. *Poster presented at the Joint ACTIRIMS-ECTRIMS Meeting, Sept 10–13, 2014, Boston, USA*

[31] Laroni A, Brogi D, Morra VB, *et al*. Safety of the first dose of fingolimod for multiple sclerosis: results of an open-label clinical trial. *BMC Neurol.* 2014;14:65.

[32] Sacca F, Puorro G, Marsili A, *et al*. Fingolimod first dose safety in clinical practice. *Mult. Scler.* 2013;19(11 Suppl. 1):270.

[33] Capobianco M, Sapio A, Malentacchi M, *et al*. No impact of current therapeutic strategies on disease reactivation after natalizumab discontinuation: a comparative analysis of different approaches during the first year of natalizumab discontinuation. *Eur. J. Neurol.* 2014.

[34] Rinaldi F, Miante S, Perini P, *et al*. Clinical and MRI outcomes in fingolimod-treated relapsing remitting multiple sclerosis (RRMS) patients previously exposed to natalizumab: An 18-month follow-up. *Mult. Scler.* 2013;19(11 Suppl. 1):249.

[35] Rossi S, Rocchi C, Studer V, *et al*. The autonomic balance predicts cardiac responses after the first dose of fingolimod. *Mult. Scler.* 2014.

[36] Cavalla P, Binello E, Masera S, *et al*. Impact of fingolimod on comorbid migraine in multiple sclerosis. *Mult. Scler.* 2013;19(11 Suppl. 1):251-252.

[37] Kira J, Fujihara K, Ueda K, *et al*. Safety and Efficacy of Fingolimod in Japanese Patients with Multiple Sclerosis: Interim Analysis Results from Post-marketing Surveillance. *Poster presented at the PAN-Asian Committee for Treatment and Research in Multiple Sclerosis (PACTRIMS), 6–8 November 2013, Kyoto, Japan*

[38] Okamoto T, Lin Y, Araki M, *et al*. Administering fingolimod to patients with multiple sclerosis. *Mult. Scler.* 2014;20(7):929.

[39] Alroughani R, Ahmed SF, Behbehani R, *et al*. Use of fingolimod in patients with relapsing remitting multiple sclerosis in Kuwait. *Clin. Neurol. Neurosurg.* 2014;119:17-20.

[40] Al-Hashel J, Ahmed SF, Behbehani R, *et al*. Real-world use of fingolimod in patients with relapsing remitting multiple sclerosis: a retrospective study using the national multiple sclerosis registry in kuwait. *CNS Drugs* 2014;28(9):817-24.

[41] Gold R, Comi G, Palace J, *et al*. Assessment of cardiac safety during fingolimod treatment initiation in a real-world relapsing multiple sclerosis population: a phase 3b, open-label study. *J. Neurol.* 2013;261(2):267-76.

[42] Cohen JA, Rosenstiel P von, Gottschalk R, *et al*. Long-term safety of fingolimod: interim evaluation of data from the LONGTERMS trial. *Poster presented at the 66th American Academy of Neurology Annual Meeting, April 26–May 3, 2014, Philadelphia, PA, USA*

[43] Rosenstiel P, Cohen J, Gottschalk R, *et al*. Long-term safety of fingolimod: Interim evaluation of data from the LONGTERMS trial. *J. Neurol.* 2014;261:S248.

[44] Jokubaitis VG, Li V, Kalincik T, *et al*. Fingolimod after natalizumab and the risk of short-term relapse. *Neurology* 2014;82(14):1204-11.

[45] Kalincik T, Horakova D, Spelman T, *et al*. Comparative efficacy of switch to natalizumab or fingolimod in active relapsing-remitting multiple sclerosis. *Poster presented at the Joint ACTIRIMS-ECTRIMS Meeting, Sept 10–13, 2014, Boston, USA*

[46] He A, Spelman, Jokubaitis V, *et al*. Comparison of fingolimod versus interferon β/glatiramer acetate as second-line therapy in active MS. *Poster presented at the Joint ACTIRIMS-ECTRIMS Meeting, Sept 10–13, 2014, Boston, USA*

[47] Giacomini PS, Butzkueven H, Cohan S, *et al*. Baseline characteristics and interim analysis results of fingolimod cohort in the world-wide Post-Approval Safety program (PASSAGE). *Poster presented at the 66th American Academy of Neurology Annual Meeting, April 26–May 3, 2014, Philadelphia, PA, USA*

[48] Maciejek Z, Wójcik-dr H, Wawrzyniak S, *et al*. Evaluation of efficacy , safety and tolerability of fingolimod in patients with the relapsing form of multiple sclerosis - 12-month observation . A preliminary report. *Neurol Neurochir Pol.* 2013;47(2):145-51.

[49] Marques IB, Correia I, Batista S, *et al*. Fingolimod after Natalizumab – Is there a rebound of MS activity? *Poster presented at the Joint ACTIRIMS-ECTRIMS Meeting, Sept 10–13, 2014, Boston, USA*

[50] Chinea AR, Estades ER, Hernández YG. Can Fingolimod reduce the occurrence of MRI proven black hole and cerebral atrophy burden in Hispanic population with Multiple Sclerosis? *Poster presented at the Joint ACTIRIMS-ECTRIMS Meeting, Sept 10–13, 2014, Boston, USA*

[51] Chinea A, Bryan J, Rodriguez L. Analysis of cardiovascular events during first dose observation and during 6 month of treatment with fingolimod in hispanic multiple sclerosis population. *Mult. Scler.* 2012;18(9):S12.

[52] Kotov S, Fedorova S, Iakushina T, *et al*. The experience of using fingolimod (Gilenya) in patients with relapsing-remitting multiple sclerosis. *Zh Nevrol Psikhiatr Im S S Korsakova* 2013;113(2 Pt 2):74-8.

[53] Klímová E, Daňová M. The course of washout period at JCV seropositive RRMS patients after natalizumab withdrawal and planned switch to fingolimod. *J. Neurol. Sci.* 2013;333(2013):e415-e416.

[54] Sempere AP, Martín-Medina P, Berenguer-Ruiz L, *et al*. Switching from natalizumab to fingolimod: an observational study. *Acta Neurol. Scand.* 2013;128(2):e6-e10.

[55] Rodríguez Vicente Y, Garrido Segovia C, Macías Cortes E, et al. Nuestra experiencia con fingolimod. *Rev. Científica la Soc. Española Enfermería Neurológica* 2014;39(1):22-28.

[56] Romera M, Fernandez-Bolanos R, Rus M, *et al*. An observational study of switching from natalizumab to fingolimod. *Mult. Scler.* 2013;19(11 Suppl. 1):463.

[57] Izquierdo G, Dinca L, Garcia-Moreno J, *et al*. Spanish experience with fingolimod in the clinical practice. *Mult. Scler.* 2013;19(11 Suppl. 1):218-219.

[58] Fernandez O, Antiguedad A, Oreja-Guevara C, *et al*. Spanish registry of patients with multiple sclerosis treated with fingolimod (GILENYA registry): Management and safety after starting treatment. *Mult. Scler.* 2013;19(11 Suppl. 1):492.

[59] Rodríguez-Antigüedad, Oreja-Guevara C, Montalban X, *et al*. Spanish Registry of patients with multiple sclerosis treated with fingolimod (GILENYA Registry): safety and effectiveness after one year on treatment. *Poster presented at the Joint ACTIRIMS-ECTRIMS Meeting, Sept 10–13, 2014, Boston, USA*

[60] Hernández-Clares R, Meca-Lallana J, Carreón-Guarnizo E, *et al*. Clinical and radiological activity during the fingolimod treatment prescribed after natalizumab. A multiple sclerosis unit experience. *Poster presented at the 66th American Academy of Neurology Annual Meeting, April 26–May 3, 2014, Philadelphia, PA, USA*

[61] Palazon B, Carreon E, Clares R, *et al*. Electrocardiogram monitoring after the first fingolimod doses. *Neurology* 2014;82(10 Suppl. 1):P2.219.

[62] Regal AR, Midaglia L, Arpín EC, *et al*. Efficacy and safety of Fingolimod in clinical practice. *Poster presented at the Joint ACTIRIMS-ECTRIMS Meeting, Sept 10–13, 2014, Boston, USA*

[63] Alcala C, Perez-Miralles F, Bosca I, *et al*. Fingolimod plus steroids prevents return to clinical activity after natalizumab withdrawal. *Mult. Scler.* 2013;19(11 Suppl. 1):449.

[64] Frisell T, Jonsson L, Nordin N, *et al*. Comparative analysis of drug discontinuation during the first year of fingolimod versus natalizumab treatment in the Swedish IMSE registry. *Poster presented at the Joint ACTIRIMS-ECTRIMS Meeting, Sept 10–13, 2014, Boston, USA*

[65] Feltelius NP, Olsson NDC. The immunomodulation and multiple sclerosis epidemiology (IMSE II) study: A Swedish nationwide pharmaco-epidemiological and genetic study focussed on long-term safety and efficacy of fingolimod (Gilenya). *Mult. Scler.* 2013;19(11 Suppl. 1):445-446.

[66] Naegelin Y, Rasenack M, Sanderson N, *et al*. An observational study evaluating disease control, safety, and immunological changes in patients with relapsing remitting multiple sclerosis switching from previous treatment with natalizumab to fingolimod (SWITCHUHBS). *Mult. Scler.* 2013;19(11 Suppl. 1):460.

[67] Czaplinski A, Metzler F, Stellmes P, *et al*. Final Results of the Swiss Post Marketing Surveillance Monitoring Quality of Life and Treatment Satisfaction in Patients with Relapsing-Remitting Multiple Sclerosis (SWISSASCENT). *Poster presented at the Joint ACTIRIMS-ECTRIMS Meeting, Sept 10–13, 2014, Boston, USA*

[68] Altunrende B, Shugaiv E, Yabalak A, *et al*. Fingolimod in relapsing remitting multiple sclerosis: Short term safety profile. *Mult. Scler.* 2014;20(7):928.

[69] Webber B, Pugh P, Whitlock C, *et al*. Management of fingolimod first dose cardiac monitoring in UK multiple sclerosis patients. *Mult. Scler.* 2013;19(11 Suppl. 1):256.

[70] Rotstein D, Malik MT, Healy B, *et al*. Clinical Predictors for Fingolimod (Gilenya) Treatment Response in Multiple Sclerosis Patients. *Neurology* 2014;82(10 Suppl.):P7.225.

[71] Rotstein D, Malik MT, Healy B, *et al*. Clinical Predictors of Fingolimod Treatment Response in Multiple Sclerosis Patients. *Poster presented at the Joint ACTIRIMS-ECTRIMS Meeting, Sept 10–13, 2014, Boston, USA*

[72] Vo K, Stazzone L, Nguyen H, *et al*. Retrospective analysis of the safety and tolerability profile of fingolimod in clinical practice. *Mult. Scler.* 2013;19(11 Suppl. 1):247.

[73] Ontaneda D, Hara-Cleaver C, Rudick R a, *et al*. Early tolerability and safety of fingolimod in clinical practice. *J. Neurol. Sci.* 2012;323(1-2):167-72.

[74] Hersh C, Hara-Cleaver C, Rudick R, *et al*. Tolerability and Efficacy of Fingolimod in Clinical Practice. *Neurology* 2013;80(Suppl. S):P01183.

[75] Miller D, Lee J-C, Hashmonay R, *et al*. Patient disability and quality of life after fingolimod initiation: The Cleveland Clinic experience. *Mult. Scler.* 2013;19(11 Suppl. 1):444.

[76] Bermel R, Xu L. Retinal effects of fingolimod in 232 patients studied with optical coherence tomography. *Mult. Scler.* 2013;19(11 Suppl. 1):431.

[77] Samuel-Cohn B, Bermel RA, Hara-cleaver C, *et al*. Comparative Tolerability and Efficacy of Dimethyl Fumarate And Fingolimod in Multiple Sclerosis. *Poster presented at the Joint ACTIRIMS-ECTRIMS Meeting, Sept 10–13, 2014, Boston, USA*

[78] Carroll C. Comparing adherence rates to disease modifying therapies for multiple sclerosis in commercial enrollees and Part D beneficiaries. *Value Heal.* 2013;16(3):A108.

[79] LaGanke C. Natalizumab Exit Strategy Experience with Fingolimod. *Poster presented at the 65th American Academy of Neurology Annual Meeting, 16–23 March 2013, San Diego, CA, USA*

[80] Schurmann P, Basra S, Awar OG, *et al*. Abnormal rhythms in patients without known cardiac disease after a first dose of fingolimod. *Mult. Scler. Relat. Disord.* 2014;3(3):408-412.

[81] Agashivala N, Wu N, Abouzaid S, *et al*. Compliance to fingolimod and other disease modifying treatments in multiple sclerosis patients, a retrospective cohort study. *BMC Neurol.* 2013;13(1):138.

[82] Edwards K, Crayton H, Calkwood J, *et al*. Patient-and physician-reported outcomes after therapy switch from interferon beta to fingolimod versus staying on interferon beta therapy. *Mult. Scler.* 2013;19(11 Suppl. 1):231-232.

[83] Hanson K a, Agashivala N, Stringer SM, *et al*. A cross-sectional survey of patient satisfaction and subjective experiences of treatment with fingolimod. *Patient Prefer. Adherence* 2013;7:309-18.

[84] Hanson K a, Agashivala N, Wyrwich KW, *et al*. Treatment selection and experience in multiple sclerosis: survey of neurologists. *Patient Prefer. Adherence* 2014;8:415-22.

[85] Salter AR, Cofield SS, Fox RJ, *et al*. Disability progression associated with transitioning treatment after 2 years of natalizumab therapy as reported by NARCOMS participants. *Poster presented at the Joint ACTIRIMS-ECTRIMS Meeting, Sept 10–13, 2014, Boston, US*

[86] Carruthers RL, Rotstein DL, Healy BC, *et al*. An observational comparison of natalizumab vs. fingolimod using JCV serology to determine therapy. *Mult. Scler.* 2014;20(10):1381-90.

[87] Bergvall N, Petrilla A a, Karkare SU, *et al*. Persistence with and adherence to fingolimod compared with other disease-modifying therapies for the treatment of multiple sclerosis: a retrospective US claims database analysis. *J. Med. Econ.* 2014;17(10):696-707.

[88] Bergvall N, Lahoz R, Reynolds T, *et al*. Healthcare resource use and relapses with fingolimod versus natalizumab for treating multiple sclerosis: a retrospective US claims database analysis. *Curr. Med. Res. Opin.* 2014;30(8):1461-71.

[89] Bergvall N, Makin C, Lahoz R, *et al*. Relapse Rates in Patients with Multiple Sclerosis Switching from Interferon to Fingolimod or Glatiramer Acetate: A US Claims Database Study. *PLoS One* 2014;9(2):e88472.

[90] Bergvall N, Makin C, Lahoz R, *et al*. Comparative effectiveness of fingolimod versus interferons or glatiramer acetate for relapse rates in multiple sclerosis: a retrospective US claims database analysis. *Curr. Med. Res. Opin.* 2013;29(12):1647-56.

[91] Repovic P, Lee D, Hamilton S, *et al*. Effect of fingolimod on macular volume and retinal nerve fiber layer thickness: A one year longitudinal study. *Neurology* 2014;82(10 Suppl. 1):1526-632X.

[92] Bergvall N, Lahoz R, Nazareth T, *et al*. Persistence with fingolimod versus dimethyl fumarate in patients with multiple sclerosis: a retrospective analysis using US open and closed data sources. *Poster presented at the Joint ACTIRIMS-ECTRIMS Meeting, Sept 10–13, 2014, Boston, USA*

[93] Hughes B, Cascione M, Freedman MS, *et al*. First-dose effects of fingolimod after switching from injectable therapies in the randomized, open-label, multicenter, Evaluate Patient OutComes (EPOC) study in relapsing multiple sclerosis. *Mult. Scler. Relat. Disord.* 2014;3(5):620-628.

[94] Hunter S, Hollander E, Miller D, *et al*. Impact of fingolimod on depressive symptoms: Post-hoc data from the EPOC trial. *Mult. Scler.* 2013;19(11 Suppl. 1):470.

[95] Fox E, Edwards K, Burch J, *et al*. Treatment Satisfaction After Switching To Fingolimod Directly From An Injectable Therapy. *Poster presented at the 66th American Academy of Neurology Annual Meeting, April 26–May 3, 2014, Philadelphia, PA, USA*

[96] Dibernardo A, Agashivala N, Meng X, *et al*. Effect of fingolimod on two subdomains of the Beck Depression Inventory-II in patients with relapsing MS. *Poster presented at 29th congress of the European Comimttee for Treatment and Research In Multiple Sclerosis, 2-5 October, Copenhagen, Denmark*

[97] LaGanke C, Wynn D, McCague K, et al. No effect on incidence of infection after therapy switch to fingolimod. J. Neurol. 2013;260:S127.
